# Latvian family physicians’ experience diagnosing depression in somatically presenting depression patients: A qualitative study

**DOI:** 10.1080/13814788.2017.1291626

**Published:** 2017-03-22

**Authors:** Maija S. Leff, Jeļena Vrubļevska, Agita Lūse, Elmārs Rancāns

**Affiliations:** ^a^ Department of Communication Studies, Riga Stradiņš UniversityRigaLatvia; ^b^ Department of Psychiatry and Narcology, Riga Stradiņš UniversityRigaLatvia

**Keywords:** Depression, unexplained somatic complaints, general practice, qualitative methods, diagnosis

## Abstract

**Background:** Depression continues to be under-diagnosed in primary care settings. One factor that influences physicians’ likelihood of diagnosing depression is patients’ presentation style. Patients who initially present with somatic symptoms are diagnosed at a lower rate and with greater delay than patients who present with psychosocial complaints.

**Objectives:** To identify the barriers preventing depression diagnosis in somatically presenting patients in an Eastern European primary care setting.

**Methods:** Thematic analysis of semi-structured interviews with 16 family physicians (FPs) in Latvia. FPs were sampled using a maximum variation strategy, varying on patient load, urban/rural setting, FP gender, presence/absence of on-site mental health specialists, and FP years of practice.

**Results:** FPs observed that a large subgroup of depression patients presented with solely somatic complaints. FPs often did not recognize depression in somatically presenting patients until several consultations had passed without resolution of the somatic complaint. When FPs had psychosocial information about the somatically presenting patient, they recognized depression more quickly. Use of depression screening questionnaires was rare. Barriers to diagnosis continued beyond recognition. Faced with equivocal symptoms that undermined clinical certainty, FPs postponed investigating their clinical suspicion that the patient had depression and pursued physical examinations that delayed depression diagnosis. FPs also used negative physical examination results to convince reluctant patients of a depression diagnosis.

**Conclusion:** Delayed recognition, the need to rule out physical illness, and the use of negative physical examination results to discuss depression with patients all slowed the path to depression diagnosis for somatically presenting patients in Latvian primary care.

KEY MESSAGES

Family physicians recognized depression in their somatically presenting patients gradually; for some physicians, suspicion was triggered not by detection of key depression symptoms, but failure to uncover organic pathology during examinations.
Psychosocial information about patients facilitated recognition.
Even when family physicians suspected depression, uncertainty encouraged the pursuit of somatic investigations.

## Introduction

Depression is amongst the most common mental disorders [1]. It is associated with disability, decreased quality of life, and increased mortality [2–4]. However, the recognition of depression in primary care settings remains sub-optimal [5,6]. To improve care, it is necessary to collect information about the barriers primary care physicians experience as they attempt to diagnose depression. Despite rich data from studies of depression in primary care in Western Europe [7,8], there still is a need for studies from Eastern Europe. The best available data suggest that under-diagnosis of depression is particularly salient for the Eastern European Republic of Latvia, where the point prevalence of depression in the general population has been measured at 6.7% but only 0.6% of the population received treatment for depression in state-funded clinics in 2010 [9,10].

The high proportion of depression patients who present with solely somatic concerns complicates diagnosing depression in the primary care clinic. According to WHO data, 69% of patients in primary care settings meeting the diagnostic criteria for depression present with somatic symptoms as their primary reason for seeking care [11]. The dominance of somatic symptoms amongst depressed patients goes beyond comorbidity. Patients attribute their symptoms to depression itself, with sizable portions indicating that somatic symptoms, such as low energy and heart palpitations, characterized their most recent bout of depression [12]. Importantly, patients presenting with somatic complaints are less likely to be diagnosed with depression or diagnosed with greater delay, than patients who present with psychosocial complaints [13,14]. Creating strategies to recognize depression in these patients could alleviate these differences. Emerging data suggests, however, that these strategies will need to be regionally specific. The likelihood that patients with depression present with somatic complaints differs by country [11], and patients from different countries attribute their somatic complaints to different root causes [15]. Culture may influence these differences. In Latvia, anthropologist Vieda Skultans has described Soviet-era conceptualizations of distress as somatically-oriented [16]. In patients’ narratives from that period ‘nerves’ (*nervi*) occupy the focal point. Nerves absorb social/political trauma, and, once damaged, cause the patient emotional unrest [16]. Bodies also communicate trauma; one individual Skultans interviewed describes suffering from ‘constant tiredness’ as a result of the stress of Soviet-era military service [17]. Skultans observed a shift away from somatic descriptions of distress amongst psychiatrists in the post-Soviet period [16]. However, it remains undocumented whether these ideas still hold in the community. In this regard, it is notable that in 2013, the Latvian Centre for Disease Prevention and Control data showed that FPs saw 4423 unique patients with the diagnosis of a mood disorder, but around 50 000 with a neurotic disorder, the most prevalent being somatoform autonomic dysfunction [18].

This study presents a qualitative analysis of the barriers encountered by Latvian family physicians as they diagnose depression amongst somatically presenting depression patients. These findings are intended to add a new regional perspective to the literature on the geographically heterogeneous phenomenon of somaticized depression [11,15]. Comparisons of data from this study with findings from other countries are made, and recommendations for changes to continuing medical education proposed.

## Methods

### Sample

The authors used a maximum variation sampling strategy [7]. Practitioners who varied along four characteristics found to influence depression care/recognition in previous qualitative studies—patient load [19], urban/rural setting [19], gender [20], and presence/absence of on-site mental health specialists—were invited to participate in the study [19]. Early participants suggested that sampling included variation in the number of years physicians had practiced family medicine; this became the fifth sampling characteristic. Information about these five characteristics was collected at the interview by questionnaire. Participants were then asked to suggest contacts who differed from them in at least one of the sampling characteristics for the next round of interviewing (a ‘snowball’ recruitment strategy) [21].

### Data collection and analysis

The initial semi-structured interview questions were developed through literature review [19,22]. As the study progressed, the authors added questions to explore the emergent theme, ‘somatic presentations of depression.’ See Table 1 for examples of interview questions. Maija Leff (ML) carried out all interviews in 2013.

**Table 1.** Initial interview guide and revised guide focusing on somatic presentations.

Sample questions from the initial interviews	Examples of added probes focusing on somatic presentations of depression
1. How often do you work with patients who have depression?2. At what point in the consultation do you start to think that a patient has depression?3. Are there any factors that delay you in diagnosing depression? Prevent you from diagnosing depression?4. What are you most concerned about when caring for patients with depression?5. What do you think are the most significant emotional and mental health problems in Latvia today?	1. At what point do you start to recognize that these (somatically presenting) patients have depression?2. Have there been any cases when you have been able to recognize depression in a somatically presenting patient during the first consultation?3. Can you describe a consultation with this type of patient?4. When does this type of patient start to recognize that they have depression?

The analysis was guided by Braun’s and Clarke’s framework for thematic analysis [23]. ML read transcripts from the first five interviews, and then split transcripts into one-line to one-paragraph segments to fracture the relevant data into distinct concepts. Segments were sorted into groups, and these became the initial inductive codes.

Agita Lūse (AL) reviewed the coding scheme and advised modifications to the interview protocol for additional interviews. Interviews concluded when codes reached saturation [24]. Jeļena Vrubļevska (JV) independently applied these codes to the final data set. ML and JV discussed all coding discrepancies until consensus was achieved. All authors contributed to the final analysis.

The Riga Stradiņš University Ethics Committee approved this study. All participants gave informed consent before participation.

## Results

### Participant demographics

Thirty-four FPs were invited to participate in the study; 16 consented to interviews with audio recording. Most were women with fewer than 2000 registered patients. One-quarter worked in a clinic with mental health specialists on-site, and about one-third worked in rural areas. On average, FPs had practiced for 15 years (range: 2–31 years) (See Table 2).

**Table 2.** Characteristics of participants’ practices, participant demographics.

Physician/practice characteristics	Number of physicianparticipants withthis characteristic
Urban practice	10
Rural practice	6
<2000 Registered Patients	14
>2000 Registered patients	2
Mental health specialist on-site	4
Mental health specialist off-site	12
Female physician	14
Male physician	2
Years practiced, range: 2–31 years	Average years practiced:15 years

### Patients rarely came to a consultation complaining of depression; somatic presentations of depression were common

An early theme in interviews was that it was unusual for patients to approach FPs complaining of depression. Nine of the 16 FPs noted that patients rarely brought up depression themselves, some indicating that even lay complaints about mood were uncommon. Instead, FPs (13 of 16 in the study) described patients presenting with somatic complaints:

FP: *They will come; complain about their heart, about dizziness, palpitations, about problems with their stomach or intestines. There are all kinds of complaints … but, now, that a patient would come and say, ‘You know, I’m having a hard time, I am depressed, I lack interest,’ that, actually I don’t recall…* (**FP34**)

Since this theme of somatically presenting depression patients was prevalent, the authors focused interviews on exploring diagnosis amongst these patients.

### Gradual recognition of depression

A common diagnostic pathway for somatically presenting depression patients centred on physical investigations. FPs (7 of 16 in the study) started by examining the organ system underlying the complaint. In this context, FPs began to suspect depression when physical examinations failed to find a cause for patients’ complaints:

Interviewer: *With somatic patients, when does it become clear (that they have depression), in the first consultation or later?*

FP: *In the first consultation, for the most part not. I examine them, and if I can’t find anything objective in the examinations, if they come again and again, and again different complaints appear, then I start to suspect that maybe it’s depression.* (**FP53**)

It was this failure to find physical disease, rather than recognition of key signs and symptoms of depression, which prompted FPs to start considering depression:

FP: *…If (the patient) walks from one physical examination to the next and everything is fine, and you can’t find anything, but (the patient) feels worse and worse, then you have to start to think if there’s not something else underneath it all.* (**FP34**)

Once this suspicion was established, FPs could proceed with next steps—for example further probing patients about stressors and changes in mood or sleeping habits. FPs also referred patients to specialists for final diagnosis after this point. However, because this pathway to the recognition of depression was gradual and required FPs to go through several rounds of physical examinations, a delay could emerge between the patient’s initial visit and their moment of diagnosis. This delay was the first diagnostic barrier identified in this study.

### Conditions for rapid recognition of depression

There were also conditions under which FPs recognized depression early in their consultations with somatically presenting patients. Most commonly (five FPs), this occurred when FPs had information about patients’ psychosocial conditions. There were three contexts in which FPs were able to access this information. FPs who lived in the same rural community as their patient, as well as those who provided care to the patient’s family, were able to use this context to ascertain *‘how relationships are, what’s happening in the family, which also helps a little to untangle the thread’* (**FP9**). FPs were also able, under certain conditions, to collect psychosocial information directly from patients. However, FPs noted that to open discussions about psychosocial issues with patients, they needed more time in consultations:

FP: *In more detailed conversations it turns out … they have lost work or their child is having trouble with school or something else like that, and then it creeps out, that the whole blame for the high blood pressure … shortness of breath … it is from nerves, that is to say, depression symptoms … but (you need) time to talk with the patient, instead of immediately sending them to get tests and all of that and saying, I am prescribing pills, you get the tests, and come tomorrow. If there is time and you talk to the patient, the diagnosis creeps out.* (**FP87**)

### Screening questionnaires

No FPs indicated that they used screening questionnaires to help recognize depression in somatically presenting patients.

### Barriers to the initiation of treatment beyond the problem of recognition

FPs’ narratives about the work they did with somatically presenting depression patients revealed that, even after recognizing depression in their patients, FPs faced further barriers to reaching a diagnosis. Specifically, FPs felt their clinical judgment did not allow them definitively to ‘rule out’ the risk of major somatic disease. Thus, even when they suspected depression, FPs tested for physical illness to avoid missing a diagnosis. Eight of 16 FPs in the study practiced ‘ruling out.’

FPs’ attempt to balance moving forward on a suspected depression case against not *‘letting something (physical) slip by’* (**FP47**) was complicated by a sense of insecurity about interpreting somatic complaints. FPs used uncertain language when discussing the meaning of patients’ complaints, proposing that patients were suffering from depression, but then interrupting themselves with the comment that there was always the possibility of physical illness:

FP: *Usually (depression patients come) with somatic complaints. In that case, something hurts. Now I am not saying that there are not organic problems as well, let us put it that way…*

Interviewer: *And then, how does the conversation go?*

FP: *The conversation, well usually it starts with the patient explaining the problem that they have. Well, in the beginning, I always examine them, whether there is not anything underneath it, some small thing that could be there, yeah?* (**FP29**)

This phenomenon of using physical examinations to resolve the insecurity around interpreting somatic complaints occurred in five interviews. FPs’ insecurity was rooted in a view that the complaints were ultimately equivocal—using clinical intuition alone, it was not possible to declare whether the complaint indicated physical or mental illness. This was relevant even in cases when the physician strongly suspected depression. One FP expressed that his inability to guarantee that he was correct when he judged a somatic complaint to signal depression meant that he pursued physical examinations if patients pushed this:

FP: *They get physical examinations, which perhaps I would not assign, you know, classically, it does not seem to me that (something’s) there. Nevertheless, usually, I tell the patient that, yeah? I try to talk with him that I do not think that it is needed, but at the same time, well the thing is that I also do not know. What if there is something there after all.* (**FP73**)

‘Ruling out’ could become a barrier to treatment of depression when it lengthened the period between FPs’ initial suspicions of depression and the initiation of depression care. FPs’ estimates for the time it took to complete the ‘ruling out’ process ranged from three weeks (**FP34**) to four months (**FP78**).

### Using physical examinations to convince patients of the depression diagnosis

There was one additional reason that FPs pursued the examinations that could extend the wait before a depression diagnosis. FPs felt that many somatically presenting depression patients were reluctant to accept a mental health diagnosis, as the patients believed that their symptoms signalled a physical illness. To convince patients that their symptoms were related to mental health, four FPs reported using negative physical exam results to ‘prove’ to the patient that the somatic illness about which they were concerned did not exist. For some FPs, issues of stigma informed how they understood patients’ reluctance to switch from a physical to a mental health diagnosis:

FP: *People do not like to consult a psychiatrist … they think that that is something bad. A person can talk about the heart, can talk about tuberculosis, can talk about gynaecology, but now to admit the thought that the problem might be on a different level … not everyone is motivated and ready for that.* (**FP34**)

## Discussion

### Main findings

This study focused on FPs’ experience recognizing and diagnosing depression in patients who came to consultations with somatic complaints. In this study, differentiating somatic complaints with a physical origin from those with a psychiatric origin was a major problem for FPs. Half of the participants reported that they initially interpreted their depression patients’ somatic complaints as somatically rooted. Even after FPs suspected that their patient suffered from depression, difficulty in concluding what lay under the somatic complaints pushed FPs to pursue physical examinations before finalizing a depression diagnosis. Beyond these challenges of differentiation, FPs also reported challenges convincing some patients that their symptoms were expressions of psychiatric morbidity. This was another reason to use physical examinations before diagnosis: the negative examination results functioned as ‘proof’ of the symptoms’ psychiatric origin. Thus, this study presents an insight into the mechanisms that may be responsible for the delayed diagnoses observed for somatically presenting depression patients [14,25].

### Strengths and limitations

By examining barriers to depression recognition in an Eastern European setting, this study adds a new regional perspective to the literature on depression recognition in primary care. The study also presents FPs’ first-hand accounts of recognizing depression amongst somatically presenting depression patients.

The limitations of the study include data collection mode and sample generalizability. Interview data contain normative statements and narrative reconstructions that cannot be considered faithful representations of practice [26]. Therefore, observational work would be required to elucidate how FPs’ mental models of depression diagnosis compare to actual clinician behaviour. Additionally, from interview data alone it is not possible to confirm that the patients discussed by the FPs were accurately diagnosed with depression. Second, we employed a purposive, non-random sampling method to describe in-depth the mechanisms by which somatic presentations of depression influence diagnosis [21]. Quantitative follow-up studies will be needed to explore how prevalent these mechanisms are in larger FP populations.

### Interpretations and implications

FPs in this study noted that when they were able to access psychosocial information about their patients, they more quickly recognized depression. This finding agrees with the existing literature [8,14,25]. Given that access to psychosocial information is key to rapid recognition of depression, researchers have suggested that physicians directly question patients about psychosocial conditions [11,14]. Interestingly, international data show that amongst patients in which depression presents with somatic symptoms, the majority attribute these symptoms to psychosocial issues [11,14]. Thus, lack of psychosocial information appears to be an issue of patient disclosure rather than patient recognition. In Latvia, national mental health survey data indicates that undisclosed psychosocial suffering is prevalent. While FPs emphasized that patients do not bring up depression, 65% of the population, when asked via survey, indicated that they felt strained, stressed and/or depressed at some point in the last month [27]. Improving FPs’ training around soliciting psychosocial information from patients, using established curricula could be an effective strategy for increasing depression recognition [28]. However, it is important to note that consultation length influences FPs’ ability to collect psychosocial information [8,22]. Latvian FPs currently consult patients for 15 to 20 minutes, the more registered patients an FP has (full-time FPs must have ≥1800), the shorter this consult time may be.

Narratives about ‘ruling out’ somatic causes of illness have also been observed in other qualitative studies [8,22]. ‘Ruling out’ is the stage of the psychiatric differential diagnosis wherein practitioners exclude an underlying somatic medical condition; our participants’ communication of uncertainty around this process is consistent with the *DSM-5 handbook of differential diagnosis’* characterization of this step as ‘one of the most important and difficult distinctions in a psychiatric diagnosis.’[29] The motivations for ‘ruling out’ in this study diverge slightly from those described in a North American setting [22]. Practitioners in that study were motivated to ‘rule out’ somatic disease because of the stigma associated with depression (‘… it is a bit threatening to the patient … so you do not want to jump to that conclusion quickly …’) and the difficulty convincing patients of their illness due to the subjective nature of depression symptoms (‘There is no real objective measure for that. It’s a subjective feeling of your emotional state’) [22, p.34]. FPs in this study also spoke of stigma and the difficulty of convincing certain patients of a depression diagnosis. Moreover, they were motivated to use ‘ruling out’ to resolve the lingering concern that they had incorrectly interpreted their patients’ somatic complaints. ‘Ruling out’ is an integral step in differential diagnosis [29]. However, qualitative studies, including this one, show that ‘ruling out’ serves additional functions, such as providing ‘evidence’ to negotiate a depression diagnosis with a patient. It is important to understand these functions. With this understanding, it may be possible to address the same purpose with a more efficient process (e.g., responding to stigma with educational materials in-clinic, rather than costly physical examinations). Nevertheless, a certain level of ‘ruling out’ will always be necessary, to identify general medical conditions that can cause morbidity and increased psychiatric symptomology [29]. In this context, guidelines which promote routine screening for depression can help FPs explore a depression diagnosis earlier [30], simultaneous with necessary physical investigations, rather than after.

Finally, the self-reported use of depression screening questionnaires was low in this study. Previous qualitative studies report similar findings, with questionnaires being used to negotiate diagnoses with patients rather than to identify cases [31]. Further work should be done to determine facilitators to screening questionnaire uptake in the Eastern European setting.

One avenue to support enhanced training and increased access to screening tools is continuing medical education (CME). In the Latvian setting, family medicine residents receive two to four weeks of psychiatric education during their three-year residency period. However, once certified, FPs are not required to take any further psychiatric coursework. In 2012 and 2015, recommendations and guidelines for diagnosing and treating depression were released in Latvia, the earlier being geared towards primary care practice [32,33]. In that period, however, no targeted training for FPs on diagnosing depression occurred. Continuing education initiatives from neighbouring countries, such as the general practitioner training component of the European Alliance against Depression (http://www.eaad.net/home/), could be adopted.

## Conclusion

Delayed recognition of depression, the need to rule out physical illness, and the use of negative physical examination results to discuss depression with patients all slowed the path to depression diagnosis for somatically presenting patients in the Latvian primary care setting.
